# New-onset headache in an elderly man with uremia that improved only after correction of hyperphosphatemia ("uremic headache"): a case report

**DOI:** 10.1186/1752-1947-5-77

**Published:** 2011-02-24

**Authors:** Sushil Razdan, KK Pandita, Vanilla Chopra, Sanjay Koul

**Affiliations:** 1 Department of Medicine, ASCOMS, Sidhra, Jammu, J&K, India; 2 Department of Anaesthesia, ASCOMS, Sidhra, Jammu, J&K, India; 3 Department of Medicine, ASCOMS, Sidhra, Jammu, J&K, India

## Abstract

**Introduction:**

New-onset headaches in the elderly are usually secondary and rarely primary. We present the case of an elderly man with recent-onset headache due to uremic hyperphosphatemia and hypocalcemia. To the best of our knowledge, this is the first case report of its kind in the literature.

**Case presentation:**

We present the case of a 70-year-old Indian man with chronic kidney disease whose new-onset headache improved only when his hyperphosphatemia and hypocalcemia were corrected. He had diffuse, dense calcification of tentorium cerebelli and falx due to hyperphosphatemia.

**Conclusions:**

This case report reinforces the importance of identifying the cause of a new-onset headache, particularly in the elderly, and treating it before blaming a tension headache or primary headache as the cause.

## Introduction

Although the prevalence of headache is reduced with age, it remains a common problem in the elderly [1]. New-onset primary headaches are a rarity in this age group [2]. Appropriate identification of secondary cause of a headache is the key to successful therapy. We present an elderly patient with chronic renal failure (CRF) with hyperphosphatemia whose new-onset headache improved only with correction of hyperphosphatemia and hypocalcaemia.

## Case presentation

A 79-year-old Indian man, who had CRF due to hypertensive nephropathy for the past five years, presented with new-onset headache for the past six months. The headache was global, of moderate to severe intensity, dull in character, persistent, and occurred daily. It had no relation to coughing, straining, or posture change. It had no diurnal variation. He had a history of occasional vomiting. For the past approximately one year, he had symptoms suggestive of restless-leg syndrome. He had received maintenance hemodialysis for more than one year. He had no history of scalp tenderness, jaw claudication, persistent fever, neck pain, persistent nasal symptoms, or dental disease. No history of any obvious trauma was found. He had received sertraline, gabapentin, sodium valproate, and topiramate for the headache, with no benefit. The headache was not relieved even by non-steroidal anti-inflammatory drugs (NSAIDs).

**Figure 1:**
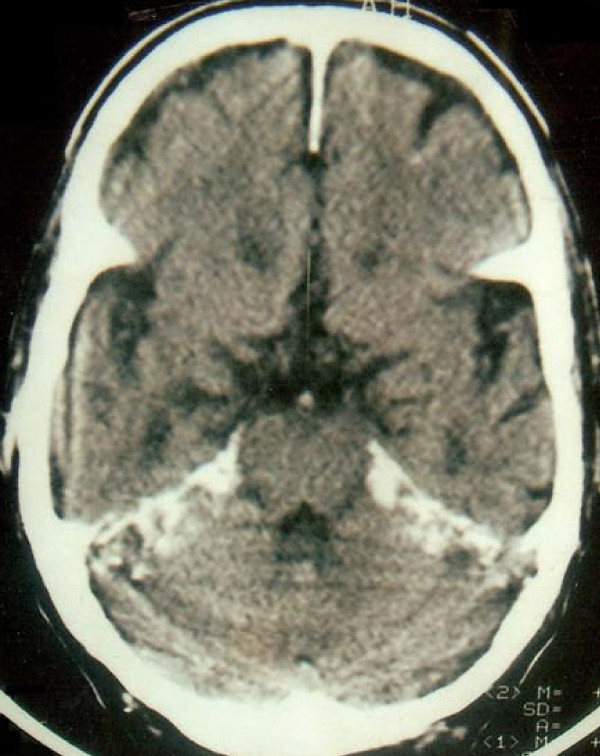
**Computed tomography scan of the head, revealing diffuse, dense calcification of the cerebellum, tentorium, and falx**.

On examination, he had mild pallor, blood pressure of 130/88 mm Hg, and a sallow complexion. The rest of his general examination and systemic examinations were unremarkable. He had no neurologic deficit. His neck was supple. Fundus examination revealed no papilloedema. Plain CT scan of his head revealed diffuse, dense calcification of the cerebellum, tentorium (Figure 1), and falx. He had a hemoglobin level ranging from 9 to 11 mg/dl (with erythropoietin; normal range (NR), 13.6 to 17.5 gm/dl), erythrocyte sedimentation rate (ESR) of 20 mm, first hour (NR, 0 to 15 mm, first hour). Blood urea level ranged from 140 to 198 mg/dl (NR, 8 to 40 mg/dl), and serum creatinine level ranged from 6 to 9.6 mg/d (NR, 0.6 to 1.2 mg/dl); serum calcium level ranged from 7.4 to 8.2 mg/dl (NR, 8.5 to 10.5 mg/dl), and the serum phosphorus level, from 6.8 to 8.0 mg/dl (NR, 2.5 to 4.5 mg/dl). His serum parathormone level was 321.1 pg/ml (NR, 15 to 68.3 pg/ml). His arterial blood gas analysis revealed no hypoxemia or hypercapnia. The rest of his investigations were unremarkable.

We continued with maintenance hemodialysis and started him on a low-phosphorus diet. Sevelamer, a phosphate binder, was started, with a dosage of 800 mg thrice daily. We uptitrated its dose to 1600 mg thrice daily. We also administered tablets containing vitamin D and calcium. His serum calcium level increased to 8.6 mg/dl, and the serum phosphate decreased to 5 mg/dl. He reported a remarkable improvement of his headache but would still have an occasional mild to moderate headache.

## Discussion

Headache with onset at an elderly age is a prominent symptom in as many as one in six persons and often has a more serious import than a headache in a younger person. Although around 40% of the elderly have tension headache, in the majority of them, a wide variety of diseases are responsible for the headache [2]. These include space-occupying lesions, temporal arteritis, trigeminal neuralgia, postherpetic neuralgia, cerebrovascular disease, hypoxia and hypercapnia, cervical spondylosis, Paget's disease, systemic disease (for example, anemia), hypocalcemia, hyponatremia, renal failure, post-traumatic headache, or severe hypertension [1-3].

In our patient, a controlled hypertension, no history of significant trauma, the absence of scalp tenderness and jaw claudication, and only a mildly accelerated erythrocytic sedimentation rate and normal serum alkaline phosphatase level rule out severe hypertension, trauma, temporal arteritis, or Paget's disease as causes of his headache. There was evidence of a space occupying lesion on CT scan of head. There was no history of neuralgias and cerebrovascular disease. Absence of response to antidepressants, various anticonvulsants, and NSAIDs would rule out, to a large extent, tension headaches and primary headaches. The most likely cause of headache in our patient was CRF and disturbances of calcium and phosphorus metabolism associated with it, because the headache improved remarkably with the correction of these metabolic abnormalities. Other conditions associated with CRF that can cause headache include severe anemia, hyponatremia, severe hypertension, and dialysis. Dialysis headaches are frontal, start within a few hours of the procedure, and are not persistent [1,4]. Our patient had controlled hypertension, mild anemia, and normal sodium levels. His headache was global, persistent, and improved despite continued dialysis. Inadequately treated hyperphosphatemia in CRF leads to secondary hyperparathyroidism and extraosseous calcification of soft tissues [5,6]. Ingested calcium is deposited in extraosseous sites, possibly because it cannot be deposited in bones [7]. Our patient had increased serum parathormone levels with diffuse dense calcification of the cerebellum, falx, and tentorium. This calcification appears to be a silent bystander, rather than a contributor to the headache, because the headache improved despite the persistence of the calcification. Nevertheless, it is a powerful clue to the possible existence of hyperphosphatemia.

## Conclusions

In conclusion, if an elderly patient presents with a headache for the first time, an underlying cause other than primary and tension headaches should be sought. In a patient with new-onset headache and a comorbidity like renal failure, comorbidity should be blamed first as a cause, identified precisely, and treated specifically before looking for other causes.

## Abbreviations

CRF: chronic renal failure; CT: computed tomography; NSAIDS: nonsteroidal anti-inflammatory drugs.

## Competing interests

The authors declare that they have no competing interests.

## Consent

Written informed consent was obtained from the patient for publication of this case report and accompanying images. A copy is available for review by the Editor-in-Chief of this journal.

## Authors' contributions

SR and KKP evaluated the patient. KKP, VC, and SK collected data, reviewed the literature, and wrote the manuscript. All authors read and approved the final manuscript.
